# Clinical evaluation of switching from immediate‐release to prolonged‐release lithium in bipolar patients, poorly tolerant to lithium immediate‐release treatment: A randomized clinical trial

**DOI:** 10.1002/brb3.2485

**Published:** 2022-02-09

**Authors:** Federica Pelacchi, Liliana Dell'Osso, Emi Bondi, Mario Amore, Andrea Fagiolini, Paolo Iazzetta, Daniela Pierucci, Manuela Gorini, Elisa Quarchioni, Alessandro Comandini, Enrica Salvatori, Agnese Cattaneo, Maurizio Pompili, SALT Study Group

**Affiliations:** ^1^ Global Medical Department, Angelini Pharma S.p.A. Rome Italy; ^2^ Department of Clinical and Experimental Medicine University of Pisa Pisa Italy; ^3^ Psychiatric Service Diagnosis and Care Papa Giovanni XXIII Hospital Bergamo Italy; ^4^ Institute of Rehabilitation and Care for Scientific Character San Martino Polyclinic Hospital University Psychiatric Clinic Genova Italy; ^5^ Psychiatry University‐Hospital of Siena Siena Italy; ^6^ Functional Unit Mental Health Adults San Giovanni di Dio Hospital Orbetello‐Grosseto Italy; ^7^ Complex Operational Unit Psychiatry Sant'Andrea University‐Hospital University of Rome La Sapienza Rome Italy

**Keywords:** bipolar disorder, lithium compounds, tremor

## Abstract

**Aim:**

The effect of switching from lithium immediate release (Li‐IR) to lithium prolonged release (Li‐PR) on lithium‐induced tremor after 1 and 12 weeks of treatment was evaluated in a randomized, multicenter, open trial, in bipolar patients from the participating sites with a tremor severity ≥2 (Udvalg for Kliniske Undersøgelser [UKU] rating scale) despite optimal lithium titration.

**Methods:**

The primary endpoint was the evaluation of tremor by means of the UKU scale after 1 week of treatment. Secondary endpoints included manic Young Mania Rating Scale (YMRS) and depressive symptoms (Montgomery–Asberg Depression Rating Scale), a global assessment of the patient's status (Clinical Global Impression), polyuria/polydipsia (UKU item 3.8) and patient‐reported outcomes.

**Results:**

Owing to difficulties in including suitable patients the enrollment phase was closed when 73 patients were randomized. Notwithstanding the lower number of patients, in the modified intention‐to‐treat population (*n* = 70) the primary endpoint was statistically significant: tremor improved after 1 week in 62.9% in Li‐PR group against 20.0% of patients in Li‐IR group (*p* = .0006; two‐tailed Fisher's exact test). The difference remained statistically significant after 4 (*p* = .0031) and 12 weeks (*p* = .0128). The same analysis performed in the PP population confirmed these results. Among the secondary endpoints, only the factor convenience of the treatment satisfaction questionnaire showed a statistically significant difference between groups. There were no apparent differences in the safety profile of the two formulations.

**Conclusions:**

This study is the first comparative documentation of a potential benefit of the prolonged‐release formulation in reducing the symptom tremor, a well‐known adverse effect of lithium therapy. Indeed, the study results should be interpreted taking into account the sample size lower than planned.

## Graphical abstract

The effect of switching from lithium immediate release (Li‐IR) to lithium prolonged release (Li‐PR) on lithium‐induced tremor after 1 and 12 weeks of treatment was evaluated in a randomized, multicenter, open trial, in 70 bipolar patients with a tremor severity ≥2 (UKU rating scale) despite optimal lithium titration. After 1 week, switching tremor improved in 62.9% in Li‐PR group against 20.0% of patients in Li‐IR group (*p* = .0006; two‐tailed Fisher's exact test). The difference remained statistically significant after 4 (*p* = .0031) and 12 weeks (*p* = .0128).

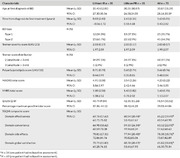

## 1 INTRODUCTION

Bipolar disorder (BD) is a lifelong episodic illness with a variable course that can often result in functional and cognitive impairment and a reduction in quality of life (Grande et al., 2016; Kessler et al., 2005). The treatment of bipolar disorder needs to be individualized and includes pharmacological agents as well as psychosocial interventions. Pharmacological treatment options to control symptoms and/or prevent illness recurrence in the management of bipolar disorder pose several difficulties for clinicians. Agents commonly used include lithium, some anticonvulsants, atypical antipsychotics, and in some cases antidepressants (National Institute for Health and Clinical Excellence [NICE], 2014).

Lithium has been a cornerstone of therapy for bipolar disorder for several decades and is effective in the management of both manic and depressive episodes (Hirschfeld et al., 2002; Grandjean & Aubry, 2009b). The effectiveness of lithium monotherapy in acute mania has been demonstrated in randomized active‐ and placebo‐controlled trials and for this reason it is a first‐line recommendation in the majority of the current clinical practice guidelines (Grandjean & Aubry, 2009b; NICE, 2014). Moreover, lithium salts have demonstrated efficacy in the prevention of mania, depression, and suicidal behavior and remain among the most commonly prescribed prophylactic medications for the maintenance phase of BD (Malhi et al., 2012). During the maintenance therapy, the abrupt cessation of lithium or a reduction in its serum concentrations can precipitate recurrence of BD symptoms in 60% to 80% of patients (Yatham et al., 2013). Continuity of treatment is thus essential.

Lithium has a narrow therapeutic window and a high incidence of troublesome side effects. The most common side effects in patients on lithium treatment are tremor, polyuria, polydipsia, weight gain, and diarrhea; these events can induce patients to low treatment compliance or to interrupt the therapy. Side effects appear to be related to peak serum levels (e.g., tremor peaks within 1/2 h of a dose). The occurrence of tremor in patients receiving lithium is well known and the clinical management of this side effect may be a significant problem both for patients and physicians. In a review study (Gelenberg & Jefferson, 1995), the pooled percentage for any complaints of tremor in patients treated with lithium was estimated to be about 27%, with individual studies showing wide variability from 4% to 65%. Lithium tremor is classified as a postural tremor; it is generally symmetric, typically produced by voluntary maintenance of a particular posture held against gravity. It is usually limited to hands or upper limbs at resting state but worsens during activities that require fine motor control such as writing or pouring water. Lithium tremor typically appears when started or titrated. Tremor can cause serious problems in patients’ daily lives, can be troublesome and socially embarrassing and might eventually cause noncompliance for treatment (Baek et al., 2014; Burgess et al., 2001). Tremor is the most frequent reason given by patients for lithium discontinuation. Even though clinicians may use beta‐blockers to treat lithium‐induced tremor, their potential side effects, together with their limited efficacy in reducing tremor, prompt for investigations to confirm that prolonged‐release lithium formulations might be devoid of tremor side effect and improve compliance (Morgan & Sethi, 2005; Shoutsuki & Terao, 2014).

Both prolonged‐release (PR) and immediate‐release (IR) preparations of lithium salts are available in the EU market. The PR formulation may allow preventing high fluctuations in lithium plasma levels and high plasma peak levels, thus providing more stable plasma concentrations compared to IR formulations. The sulfate salt of the PR formulation shows a pH‐independent solubility (unlike carbonate and citrate salts in the IR formulation) that may contribute to reduce interindividual and intraindividual variability in gastro‐intestinal absorption. Such variability is usually high and may favor lithium adverse effects or intoxication, despite the regular monitoring of lithium blood levels. Moreover, some lithium‐related adverse events appear to be a function of the rate of increase of serum lithium concentration, which has implications regarding the type of lithium formulation prescribed for a patient (Grandjean & Aubry, 2009a). Furthermore, the expected better safety profile of the PR tablets due to the reduced fluctuations in lithium plasma concentrations, and the potential improvement of the patient's compliance related to the fewer daily administration, might represent advantages over the IR formulations.

In a previous observational study, patients who switched from multiple daily administrations of lithium IR to once‐daily lithium PR declared their preference for the PR preparation over traditional one for its better tolerability and ease of administration. The occurrence of side effects in fact was significantly reduced among patients receiving the PR versus IR lithium preparation (Durbano et al., 2002).

Engagement and development of a therapeutic alliance are important in any lifelong disorder that needs long‐term adherence (Grande et al., 2016). The effectiveness of interventions aimed at increasing adherence to therapy might be helpful. Consequences of nonadherence can be serious leading to poor outcomes, worsening of the quality of life, functional impairment, and increased risks of relapse, rehospitalization, and suicidality (McIntyre & Calabrese, 2019).

Despite the fact that a prolonged‐release formulation has been on the market in several European countries since several years, there is a lack of well‐controlled clinical studies documenting the effectiveness and safety of Li‐PR in comparison to the plain formulation (Girardi et al., 2016; Martinotti et al., 2018; Nolen et al., 2019).

The present study was designed to compare the effect on tremor and other adverse effects (e.g., polyuria and polydipsia) of the switch from lithium carbonate IR formulation ((Carbolithium® immediate‐release 150 mg and 300 mg capsules, TEVA) to lithium sulfate PR formulation (Lithiofor^®^ prolonged‐release 660 mg tablets, Vifor SA / Resilient™ 660 mg prolonged‐release tablets, Angelini S.p.A), in patients affected by bipolar disorder who showed to be poorly tolerant to the lithium IR treatment.

## 2 PATIENTS AND METHODS

The study was approved by the Italian Competent Authority and by the Ethics Committees of each of the participating centers. The study was conducted according to GCP, ethical principles of the Declaration of Helsinki, and the applicable regulatory requirements. All patients gave their written informed consent before entry into the study.

The study was a multicenter, parallel group, randomized, open, and assessor blind. The study duration was 13 weeks and included a 1‐week screening period, at the end of which patients fulfilling the inclusion and exclusion criteria were randomized to one of the two treatment groups: Lithium PR (660 mg tablets) or Lithium IR (150 mg and 300 mg capsules). The choice of the open‐label design was due to the difficulties in masking the study medications. Moreover, a double‐blind, double‐dummy design was deemed unfeasible since lithium IR is used in different strengths.

The study was carried out in out‐patients recruited from six Italian psychiatric centers. Those eligible for inclusion were out‐patients of both genders aged 18–65, who fulfilled DSM‐5 criteria for Bipolar Disorder I or II (with or without rapid cycling), under optimized treatment with lithium immediate release and with a tremor severity ≥2 (single item of the Udvalg for Kliniske Undersøgelser [UKU] side‐effect rating scale) for at least 4 weeks prior to the screening visit, confirmed at baseline. Additional inclusion criteria were: a score ≤10 on the Montgomery–Asberg Depression Rating Scale (MADRS) and a score ≤12 on the Young Mania Rating Scale (YMRS) at screening and baseline visits; ability to understand the study procedures and to comply with protocol requirements. Main exclusion criteria were: schizophrenia, psychotic and schizoaffective disorders, unipolar depression, concomitant organic mental disorder or intellectual disability, history of dementia or cognitive disorders, any neurodegenerative diseases; pharmacological treatments affecting tremor, except for some pharmacological classes whose effects on tremor were stable over time and which were taken for at least 2 months before the screening visit (i.e., beta‐blockers); patients at risk for suicidal behavior; drug/alcohol abuse; clinically significant abnormalities on physical examination, vital signs, ECG, laboratory tests, prior to screening visit.

Patients randomized to lithium IR had to take orally 300–1800 mg daily divided into 2–6 doses while patients randomized to lithium PR had to take orally one tablet once or twice daily (one tablet in the morning and one tablet in the evening) or two tablets in a single dose (two tablets in the evening). At each visit the investigator had to determine the correct dosage of the study drug according to patient's condition, lithium serum/plasma levels, and his/her judgment. Serum lithium concentrations were monitored at each scheduled visit (12 h post dose). Follow‐up visits were performed after 1, 2, 4, 8, and 12 weeks from randomization.

The primary objective of the study was the evaluation of the change in the lithium‐induced tremor when switching from lithium IR to lithium PR formulation. The secondary objectives included the assessment of: (a) the effect on the lithium‐induced tremor up to 12 weeks, (b) side effects such as polyuria/polydipsia, (c) manic and depressive symptoms, (d) patient's treatment satisfaction, (e) quality of life, (f) Clinical Global Impression evaluation (CGI), and (g) safety when switching from a lithium immediate release (Li‐IR) to a lithium prolonged release (Li‐PR).

### 2.1 Assessment of efficacy

The evaluation of tremor was carried out at screening and baseline and after 1, 4, and 12 weeks of treatment by means of the UKU side effects rating scale for the registration of unwanted effects of psychotropics (Lingjaerde et al., 1987). The choice to adopt the single item 2.5 of the UKU scale for the assessment of tremor was led by the lack of specific instruments focused on this symptom. The primary endpoint was evaluated as the proportion of patients with an improvement in tremor assessed by a single item (2.5 tremor) of the UKU rating scale after 1‐week treatment period compared to baseline, improvement being defined as a difference ≥1 between scoring at baseline and scoring after 1 week of treatment. The timing for the assessment of the primary endpoint was chosen considering that a treatment period of 1 week with a PR preparation of lithium is considered adequate to reach stable lithium plasma levels. To enhance the relevance of this clinical measurement, the same efficacy assessment was evaluated as secondary endpoint at 4 and 12 weeks.

The secondary efficacy assessments were the following: (i) the effect on the lithium‐induced tremor up to 12 weeks; (ii) the evaluation of the changes in manic and depressive symptoms from baseline to 1, 4, and 12 weeks, as assessed by the YMRS (Young et al., 1978) and by the MADRS (Montgomery & Asberg, 1979); (iii) a global assessment of the patient's status (CGI) (APA, 1976) at 1 and 12 weeks; (iv) the evaluation of the changes in patient's satisfaction to the assigned treatment (treatment satisfaction questionnaire, TSQM) and quality of life (Quality of Life Enjoyment and Satisfaction Questionnaire‐Short Form, Q‐LES‐Q‐SF) from baseline to 1 and 12 weeks of treatment; (v) the proportion of patients with an improvement in polyuria/polydipsia (single item 3.8 of the UKU scale) assessed at 1, 4, and 12‐week treatment periods compared to baseline, improvement being defined as a difference ≥1 between scorings.

A blinded assessor performed all the outcome evaluations to prevent bias due to the open‐label condition of the trial.

Patients were asked to rate their satisfaction with treatment by filling in the patient's treatment satisfaction questionnaire (TSQM, version II). The 14‐item TSQM is a reliable and valid instrument to assess patients' satisfaction with medication, providing scores on four subscales—side effects, effectiveness, convenience, and global satisfaction (Atkinson et al., 2005, 2004). Quality of life was assessed by means of the Q‐LES‐Q‐SF a reliable and valid clinical instrument for the assessments of quality of life (Endicott et al., 1993; Mauri et al., 2008).

### 2.2 Assessment of tolerability

Safety assessments consisted of: (i) the assessment of polyuria/polydipsia (single item 3.8 polyuria/polydipsia of the UKU side‐effect rating scale) at baseline and after 1, 4, and 12 weeks of treatment, (ii) collection of lithium plasma/serum levels, (iii) all adverse events (AEs) and serious adverse events (SAEs), with their severity and relationship to study drug, and (iv) assessments of vital signs throughout the study. Monitoring of hematology, blood chemistry, and urinalysis performed at study centers, physical conditions and ECG were also performed at screening and at week 12. Changes in concomitant medications were collected and analyzed. Serum lithium concentrations were monitored at each scheduled visit (after 12 h post dose) and assessed as plasma or serum lithium concentrations, according to the standard methods used at the local laboratory of the centers.

### 2.3 Statistical methods

#### 2.3.1 Sample size determination

Aim of the study was to demonstrate an improvement in the proportion of patients with lithium‐induced tremor when switching from lithium IR to lithium PR. A sample of 110 evaluable patients (55 per treatment group) was considered to be sufficient to detect a clinically important difference of 20% between groups in the item 2.5 tremor of the UKU side‐effect rating scale after 1 week compared to baseline (expected improvement 5% in the Li‐IR group, and 25% in the Li‐PR one) using Fisher's exact test with a power of 80% and a 5% two‐sided significance level (nQuery Advisor). Considering a drop‐out rate of patients equal to 20%, a total number of 138 patients (69 per treatment group) had to be enrolled.

#### 2.3.2 Statistical analysis methods

Descriptive statistics was applied. Mean with standard deviation and 95% confidence intervals (CI) for variables subjected to statistical inference were calculated for quantitative variables. For qualitative variables, counts and percentages and 95% confidence interval (Wald method) were provided. All baseline characteristics were summarized by groups and overall, for the modified intention‐to‐treat (ITT) population.

The primary endpoint was analyzed using a Fisher's exact test to compare the proportion of patients with an improvement on item 2.5 tremor in the two treatment groups after 1 week, at a statistical significance of 0.05 (two sided).

For the secondary endpoints tremor (UKU item 2.5) and polyuria/polydipsia (UKU item 3.8), the same analysis was performed to compare the proportion of improved patients after 4 and 12 weeks of treatment compared to baseline. The changes from baseline on the MADRS and YMRS total scores at 1‐, 4‐, and 12‐week treatment period, and the changes from baseline for each subscale (effectiveness, side effects, convenience, and global satisfaction) of the TSQM at 1‐ and 12‐week treatment period were assessed using an analysis of covariance (ANCOVA) for repeated measurements, with the baseline as a covariate, or an analysis of variance (ANOVA) for repeated measurements if the parallelism test was statistically significant. Quality of life (Q‐LES‐Q‐SF) and CGI were analyzed by descriptive statistics.

The raw data of the Q‐LES‐Q‐SF and TSQM questionnaires were transformed according to the pertinent instructions.

The Last Observation Carried Forward (LOCF) method was implemented to handle missing data in the m‐ITT population. In calculation of percentages, patients with missing data were not considered.

All prior and concomitant medications were coded according to the Anatomical Therapeutic Chemical (ATC) classification system and summarized with descriptive statistics by active compound within each treatment group.

Adverse events were coded according to version 18.0 of the MedDRA dictionary and summarized by system organ class category (SOC) and preferred category term (PT) by frequency of occurrence within each treatment group.

The analyses for the primary and secondary efficacy evaluations were conducted on the m‐ITT population which consisted of all randomized patients who took at least one dose of study medication and had an evaluation of the primary endpoint (tremor) both at baseline and at 1 week of treatment. The analyses were repeated on the Per‐Protocol (PP) population, defined as all patients in the m‐ITT with no major protocol deviations and a treatment compliance ≥80% from baseline to 1‐week treatment period. The inclusion or exclusion from the statistical analysis of the protocol deviators with respect to the fulfillment of the inclusion/exclusion criteria, patient retention, adherence to visits timelines and adherence to protocol requirements, were decided during the pre‐database lock review meeting.

The Safety Population (SP) included all randomized subjects who took at least one dose of the study treatments.

## 3 RESULTS

Patients enrolment in the study started in March 2017; due to the low enrolment rate, the Sponsor decided to halt the trial at the end of June 2019, without reaching the sample size required by the protocol. Indeed, a few months after the study started, the PR lithium formulation became available on the Italian market with the brand name Resilient™, with a consequent relevant impact on the enrolment rate. A total of 85 patients were included in the screening phase, of which 73 were randomized: 36 allocated to the Li‐IR arm and 37 to the Li‐PR arm. Reasons for not being randomized were patient's request (four cases) and screening failure patients (eight cases). Three cases were excluded from the m‐ITT population: one in the Li‐IR group, owing to the occurrence of an exclusion criterion and two in the Li‐PR group (one patient never took the study medication and one did not perform week 1 assessments), leaving 70 patients in the m‐ITT population (primary efficacy population).

The PP population consisted of 50 patients. Main reasons for exclusion were nonevaluable compliance or compliance <80% and intake of not allowed concomitant medications.

One patient was randomized but did not take any dose, leaving 72 patients in the Safety population.

**FIGURE 1:**
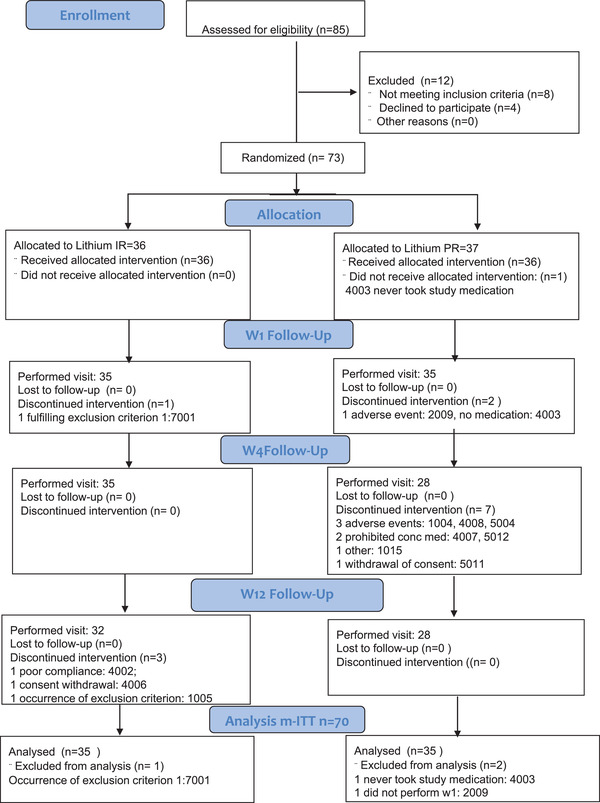
Patient disposition at the different visits

Thirty‐two and 27 patients in the Li‐IR and Li‐PR arm, respectively, completed the 12‐week treatment period. Patient disposition is displayed in Figure 1.

Overall compliance to study medication was ≥80% in 91.7% and 94.5% of patients in the LI‐IR and in the Li‐PR group, respectively. For the Li‐IR treatment group plasma/serum lithium concentrations (mEq/l) ranged from 0.32 (300 mg/day) to 0.60 (750 and 900 mg/day); for Li‐PR from 0.43 (660 mg/day) to 0.72 (1320 mg/day).

### 3.1 Sample characteristics

The clinical characteristics of patients included in the m‐ITT are presented in Table 1. The 61.4% of patients were females, with slightly more females in the Li‐IR group (65.7% vs. 57.1%).

**TABLE 1:** Baseline clinical characteristics of the randomized patients: M‐ITT population

Characteristic		Lithium IR *n* = 35	Lithium PR *n* = 35	All *n* = 70
Age at first diagnosis of BD	Mean (± SD)	31.43 (12.01)	30.31 (10.87)	30.87 (11.39)
	95% CI	27.30;35.56	26.58;34.05	28.16;33.59
Time from diagnosis to first treatment (years)	Mean (± SD)	0.83 (2.60)	2.43 (6.55)	1.63 (5.01)
	95% CI	−0.06;1.72	0.18;4.68	0.43;2.82
BD type	*N* (%)			
Type 1		12 (34.3%)	13 (37.1%)	25 (35.7%)
Type 2		23 (65.7%)	22 (62.9%)	45 (64.3%)
Tremor severity score (UKU 2.5)	Mean (± SD)	2.03 (0.17)	2.03 (0.17)	2.03 (0.17)
	95% CI	1.97;2.09	1.97;2.09	1.99;2.07
Tremor score distribution	*N* (%)			
2 (amplitude < 3 cm)		34 (97.1%)	34 (97.1%)	68 (97.1%)
3 (amplitude > 3 cm)		1 (2.9%)	1 (2.9%)	2 (2.9%)
Polyuria/polydipsia score (UKU 3.8)	Mean (± SD)	0.71 (0.79)	0.60 (0.74)	0.66 (0.76)
	95% CI	0.44;0.99	0.35;0.85	0.48;0.84
MADRS total score	Mean (± SD)	4.91 (3.08)	3.54 (3.26)	4.23 (3.22)
	95% CI	3.86;5.97	2.42;4.66	3.46;5.00
YMRS total score	Mean (± SD)	1.80 (2.08)	1.40 (1.82)	1.60 (1.95)
	95% CI	1.08;2.52	0.78;2.02	1.13;2.07
Q‐LES‐Q‐SF	Mean (± SD)	40.74 (9.84)	42.35 (11.26)	41.54 (10.52)
Percentage maximum possible total score	95% CI	37.36; 44.12	38.42;46.28	39.01;44.06
TSQM composite score	Mean (± SD)			
Domain effectiveness	95% CI	69.76 (17.63) 63.71;75.82	60.54 (20.44)^a^ 53.41;67.67	65.22 (19.49)^b^ 60.54;69.90
Domain convenience		66.98 (18.62) 60.59;73.38	59.15 (26.34)^a^ 49.96;68.34	63.12 (22.93)^b^ 57.62;68.63
Domain side effects		78.81 (17.31) 72.86;84.76	77.94 (20.40)^a^ 70.82;85.06	78.38 (18.76)^b^ 73.87;82.89
Domain global satisfaction		70.71 (15.83) 65.28;76.15	60.78 (20.47)^a^ 53.64;67.93	65.82 (18.81)^b^ 61.30;70.34

^a^ *N* = 34 (one patient had no baseline assessment).

^b^ *N* = 69 (one patient had no baseline assessment).

The two treatment groups were satisfactorily homogeneous in terms of diagnostic characteristics and baseline disease severity. About 65% of patients had a diagnosis of type 2 BD, last episode being depressive in almost all type 2 patients and in 44% of type 1 patients. A previous suicide attempt was present in 23% of cases in both groups. All patients were euthymic at inclusion in the study.

Anxiety was the most frequently associated psychiatric disturbance (62.5% and 66.7% for Li‐IR and Li‐PR, respectively). Previous psychotropic treatments were taken by 37% of patients in both groups.

Mean lithium daily dose pre‐randomization was 710.29 (±196.48) mg/day in the Li‐IR treatment group and 714.71 (±195.61) mg/day in the Li‐PR group and corresponding mean lithium serum concentrations were 0.51 (±0.20) mEq/l and 0.57 (±0.16) mEq/l, respectively.

### 3.2 Efficacy results

Primary and secondary efficacy results are described in Tables 2 and 3.

**TABLE 2:** Primary and main secondary efficacy results: M‐ITT population

Efficacy endpoints		Lithium IR	Lithium PR	*p* value
Absolute and percent frequencies of patients with improvement in tremor (UKU 2,5)	*n* (%) ‐ 95% CI			
Week 1		7 (20); 6.75/ 33.25	22(62.9);46.85/78.86	.0006**
Week 4		17 (48.6); 32.01/ 65.13	24(85.7); 72.75/98.68	.0031
Week 12		20 (64.5); 47.67/ 81.36	25(92.6); 82.71/100	.0128
Absolute and percent frequencies of patients with improvement in polyuria/polydipsia (UKU 3.8)	*n* (%) ‐ 95% CI			
Week 1		6 (17.1); 4.66/29.63	1 (2.9); 0.00/8.38	.1060**
Week 4		6 (17.1); 4.66/29.63	6 (21.4); 6.23/36.63	.7523
Week 12		7 (22.6); 7.86/37.30	6 (22.2); 6.54/37.90	1.0000
MADRS total score mean changes	Mean (± SD);95% CI			
Week 1		0.17 (3.80); −1.13/1.48	0.31 (3.38); −0.85/1.47	.0234**
Week 4		1.40 (5.40); −0.46/3.26	0.61 (4.59); −0.48/1.71	.8828
Week 12		0.34 (5.54); −1.56;2.24	0.71 (4.73); −0.41/1.84	.6803
YMRS total score mean changes	Mean (± SD);95% CI			
Week 1		−0.23 (1.70); −0.81/0.36	1.46 (1.70); 0.87/2.04	.0584**
Week 4		0.03 (2.27); −0.75/0.81	−0.31 (1.59); −0.86;0.23	.4655
Week 12		1.20 (2.03); 0.50/1.90	−0.14 (1.59); −0.69/0.40	.8220
TSQM mean changes Effectiveness	Mean (± SD); 95% CI			
Effectiveness				
Week 1		−4.52 (17.07); −10.39/1.34	2.45 (20.47); −4.69/9.59	.0643**
Week 12		−0.95 (17.12); −6.83/4.93	−2.94 (26.02); −12.02;6.14	.9090
				.5554
Convenience				
Week 1		0.16 (12.09); −3.99/4.31	16.01 (25.36); 7.17/24.86	.7875**
Week 12		1.27 (16.67); −4.46/7.00	18.46 (31.83); 7.36/29.57	.4744
				.0012
Side effects				
Week 1		1.43 (17.21); −4.48/7.34	3.92 (21.54); −3.59/11.44	.7133**
Week 12		4.52 (22.63); −3.25/12.30	5.15 (18.24); −1.22/11.51	.9966
				.7446
Global satisfaction				
Week 1		−5.95 (17.22); −11.87/−0.04	6.13 (20.95); −1.18/13.44	.2778**
Week 12		−2.14 (15.04); −7.31/3.02	5.15 (22.94); −2.86;13.15	.7872
				.2741

*Note*: UKU rating scale W1: *n* = 35 Li‐IR and 35 Li‐PR; W4: *n* = 35 Li‐IR and 28 Li‐PR; w12: *n* = 31 Li‐IR and 27 LI‐PR. The Last Observation Carried Forward (LOCF) was used to handle missing data.

*Abbreviations*: MADRS, Montgomery–Asberg Depression Rating Scale; TSQM, patient's treatment satisfaction questionnaire; YMRS, Young Mania Rating Scale.

* Two‐tailed Fisher's exact test.

** *p*‐values are from Interaction time by group, among times, and between groups terms from ANCOVA/ANOVA.

**TABLE 3:** Additional secondary efficacy results: M‐ITT population

Efficacy endpoints		Lithium IR	Lithium PR
Q‐LES‐Q‐SF percentage maximum possible total score changes	Mean (± SD);95% CI		
Week 1		1.49 (6.50); −0.75/3.72	−0.73 (7.22); −3.29/1.83
Week 12		1.32 (9.47); −2.15;4.80	0.50 (11.13); −4.00/5.00
CGI			
Week 1			
Improved	*n* (%)	8 (58.1)	19 (54.3)
No change		24 (68.6)	14 (40)
worsened		3 (8.6)	2 (5.8)
Week 12			
Improved		18 (58.1)	19 (70.3)
No change		11 (35.5)	5 (18.5)
worsened		2 (6.5)	3 (11.1)

*Abbreviations*: GI, clinical global improvement; Q‐LES‐Q‐SF, Quality of Life Enjoyment and Satisfaction Questionnaire‐short Form.

In the m‐ITT, after 1 week of treatment an improvement in tremor (assessed by item 2.5 tremor of the UKU side‐effects rating scale) versus baseline was achieved in 7 (20.0%) patients out of 35 in Li‐IR treatment group against 22 (62.9%) patients out of 35 in Li‐PR. This result was statistically significant (*p* = .0006; two‐tailed Fisher's exact test) and is corroborated by the results of the same analysis in the PP population. In the PP population, tremor improved in four (17.4%) patients out of 23 in Li‐IR treatment group against 15 (55.6%) patients out of 27 in Lit‐PR (*p* = .0084).

The difference between groups turned out to be statistically significantly different also after 4 weeks (17 out of 35 [48.6%] of patients in Li‐IR vs. 24 out of 28 [85.7%] of patients in the Li‐PR group [*p* = .0031]), as well as after 12 weeks (20 out of 31 [64.5%] of patients in Li‐IR vs. 25 out of 27 [92.6%] of patients in Li‐PR treatment group [*p* = .0128]). There was a progressive increase in the number of patients with an improvement in tremor over time in the Li‐IR group while the number of improved patients in the Li‐PR tended to remain stable.

In the PP population the difference was statistically significant after 4 weeks (*p* = .0145) but not after 12 weeks (*p* = .2317).

The symptom polyuria/polydipsia improved after 4 weeks in 6 (17.1%) patients out of 35 in the Li‐IR group against 6 (21.4%) patients out of 28 in the Li‐PR group (*p* = .7523; two‐tailed Fisher's exact test). After 12 weeks, polyuria/polydipsia improved in 7 (22.6%) patients out of 31 in Li‐IR treatment against 6 (22.2%) patients out of 27 in Li‐PR treatment (*p* = 1.0000).

**FIGURE 2:**
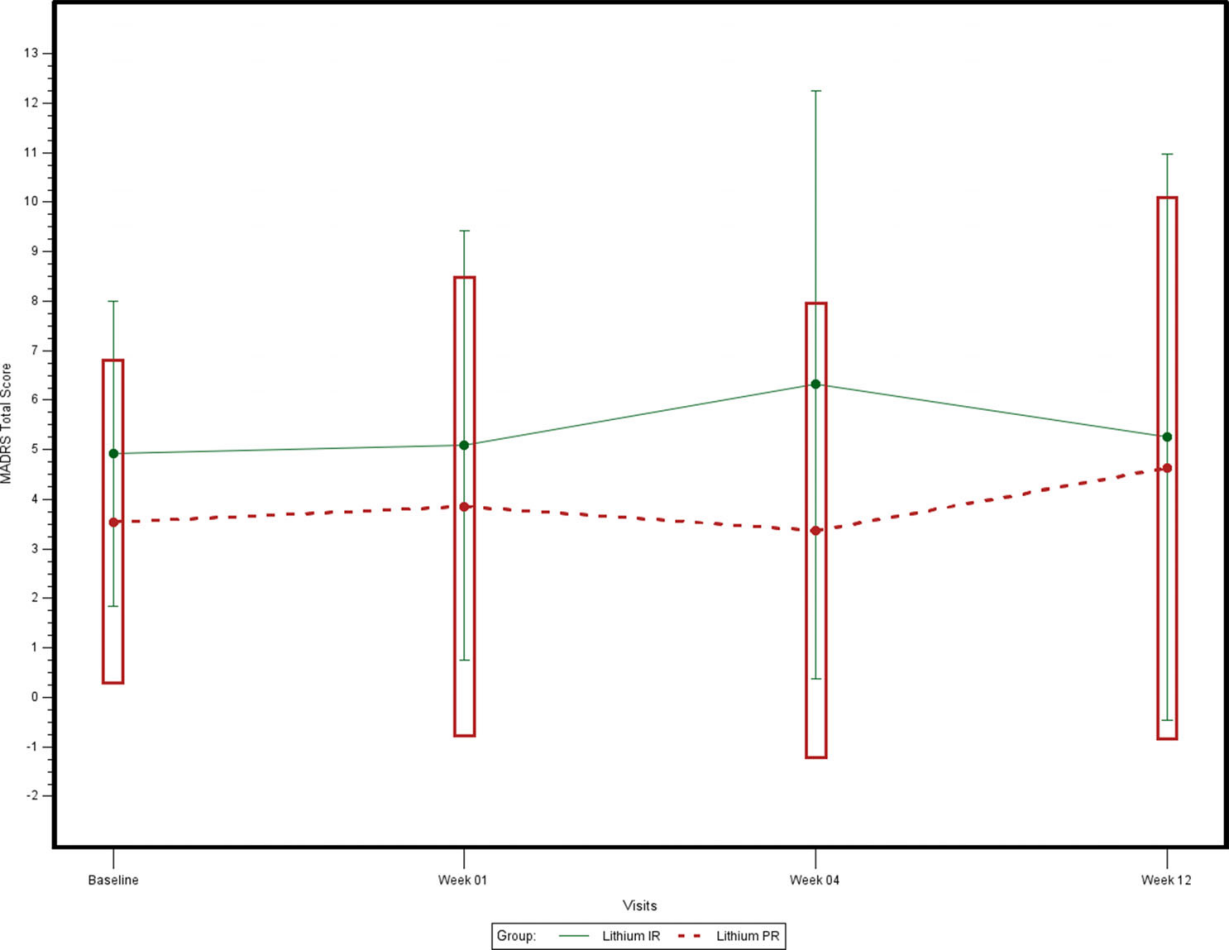
Montgomery–Asberg Rating Scale. Changes of the mean total score from baseline at weeks 1, 4, and 12, by treatment group ‐ modified ITT ‐ Last Observation Carried Forward

The evaluation of the changes in depressive symptoms (MADRS) from baseline after 1, 4, and 12 weeks of treatment for the m‐ITT showed a different qualitative pattern of the three changes between the two treatment groups (statistically significant interaction “Time by treatment”); indeed, in the Li‐IR treatment there were three increasing changes whereas in the Li‐PR treatment there was an increasing change followed by a decreasing one and by an increasing change (Figure 2, Table 2). So, the interaction significance appears due mainly by chance rather than a different expression of the two treatments. Furthermore, the changes are not correlated with the baseline values (not statistical significance for the covariate on the “Between” and “Within Subjects Effect”).

Otherwise, the mean values of changes in manic symptoms (YMRS) over time (baseline, 1, 4, and 12 weeks) were similar (interaction “Time by treatment” statistically significant) (Table 2) with not statistically significant difference between the two treatment groups. In addition, the changes were correlated with the baseline values (statistical significance for the covariate on the “Between” and “Within Subjects Effect”).

The changes over the time of the TSQM domains effectiveness, side effects, and global satisfaction did not show significant differences over time and between the treatment groups. Otherwise, for the domain convenience the difference between mean changes of the values over time turned out to be statistically significant (*p* = .0012), being greatly increased in the Li‐PR treatment.

### 3.3 Safety results

The treatment groups did not differ in terms of occurrence of adverse events, which was overall low and expected for this class of drugs and indication. At least one AE was reported in 18 (50%) and 21 (58.3%) patients in the Li‐IR and Li‐PR group, respectively, and at least one treatment‐related AE occurred in 9 (25%) patients in both groups. The most frequently reported events (>10%) were psychiatric disorders (13.9% Li‐IR; 22.2% Li‐PR): anxiety (two and three cases, in the Li‐IR and Li‐PR group, respectively; bipolar disorder (worsening of, two cases in the Li‐PR group); depression (one case in each group), insomnia and psychiatric symptoms (two cases each, in the Li‐IR group); depressed mood, elevated mood and panic attack (one case each, in the LI‐PR group).

One single death in the study (suicide) in the Li‐PR group was judged not related to the study medication by the investigator who attributed the event to the difficulties in interpersonal relationship and to the impact of life events which caused distress and ultimately suicide. Four additional serious AE occurred in three patients, two in the Li‐PR group (one possibly related, one not related) and one in the Li‐IR group (not related). Five patients (one of whom completed the final visit) discontinued the study due to the occurrence of adverse events, all in the Li‐PR group. This result turned out to be borderline statistically significant (*p* = .0539). However, it should be noted the only one event (anxiety) was judged as possibly related to the study medication. Therefore, we considered this finding an observation by chance.

From the extensive safety evaluations collected in the study (laboratory tests, vital signs, and ECG evaluation) there was no indication of potential safety issues. The safety profile of the PR formulation did not differ from the one of the IR formulation.

## 4 DISCUSSION

To our knowledge, this study is the first randomized, controlled, comparative documentation of a potential benefit of the prolonged‐release lithium formulation in reducing the severity of tremor, a well‐known adverse effect of lithium therapy, in bipolar disorder patients, when shifting from an immediate‐release formulation.

According to the scientific literature, several potential advantages of the PR lithium formulations over the IR formulations in the treatment of bipolar disorder were reported. Such reports pointed to more stable lithium serum concentrations while reaching the effective interval and, on the long term, a reduced risk of some peak‐related adverse events and a more convenient dosing regimen that may also improve adherence to therapy (Girardi et al., 2016; Martinotti et al., 2018; Nolen et al., 2019).

Despite the fact that lower than planned patients were enrolled in the study, results seem to confirm the literature data. The analysis of the primary endpoint allowed to confirm the benefit of switching from an immediate to a prolonged‐release formulation of lithium in terms of improvement of the symptom tremor. Tremor improved after 1 week and the benefit was maintained up to week 12, although the size of the difference between the two formulations decreased over time as a result of an increase in the number of patients with an improvement in tremor in the Li‐IR group. Indeed, this could be expected as it is well known that lithium‐induced tremor tends to decrease over time for immediate‐release formulations of lithium (Schou et al., 1970).

Lithium tremor is generally related to dose and lithium blood levels (Baek et al., 2014). Nevertheless, in this study, the improvement in tremor did not seem to be related to lower plasma/serum lithium levels in the Li‐PR since there were no noticeable differences in the mean plasma/serum lithium concentrations between the two groups (data not shown). Rather, the observed differences between groups may be related to different peak concentrations and not to the overall exposure. Indeed, registrative pharmacokinetic studies documented the slower increase in serum lithium concentrations and lower peak serum concentration values reached with the lithium sulfate PR formulations. However, since peak concentrations were not measured in this study, the correlation was not documented.

A raised observation to the study results was that assessing tremor at different times after each dose, as well as measuring lithium levels at different times, could help determine the relationship between tremor, dosage, and formulation.

Although we agree with this observation, it should be noticed that this was a Phase IV trial and the assessments were performed once during the day according to the routine clinical practice, at baseline and at all scheduled visits. In fact, according to the disease severity, patients included in this clinical trial were not hospitalized, thus it would have been not feasible to assess tremor at different times. To avoid bias, tremor assessment was recorded by a blinded assessor, at the same time of the day at each visit.

The switch from an IR to a PR lithium formulation did not affect the efficacy of the treatment: the two groups showed similar mean values of changes over time for the MADRS and the YMRS. This is expected because, even if prolonged‐release formulations provide more stable, consistent serum drug concentrations than immediate‐release formulations, overall exposure is essentially equivalent over the dosage interval and with long‐term administration (Grandjean & Aubry, 2009a).

The analysis of the TSQM factors effectiveness, side effects, and global satisfaction did not disclose statistically significant differences between groups. This could be explained by the fact that misattribution of symptoms as side effects of the mood stabilizers with symptoms of disease is frequent. Indeed, patients’ perception of apprehension of side effects, as opposed to the actual presence of side effects, in the most severe cases may contribute to nonadherence (Burgess et al., 2001). Instead, the TSQM factor convenience was greatly increased in the Li‐PR treatment. This could be particularly important as the use of lithium PR formulations administered once daily has been suggested as one strategy (along with dosage reduction and combination therapy) to reduce nonadherence (Gitlin, 2016). Indeed, in an observational study in 47 patients who were switched from multiple daily administration of lithium IR to once daily administration of a PR carbolithium formulation, a preference for better tolerability and ease of administration of lithium PR over lithium IR was reported (Durbano et al., 2002).

From the extensive safety evaluations collected in the study there was no indication of potential safety issues when switching from an IR to a PR lithium formulation. In the management of bipolar disorder, the primary goals are to ensure the safety of the patient and to achieve clinical and functional stabilization with minimum adverse effects, as this is a lifelong disease.

The study results should be interpreted with caution, considering the lower than planned sample size and the relatively short observation period. Further long‐term clinical data from larger patients’ cohorts may be needed to corroborate the above results.

## CONFLICT OF INTEREST

Federica Pelacchi, Daniela Pierucci, Manuela Gorini, Elisa Quarchioni, Alessandro Comandini, Enrica Salvatori, Agnese Cattaneo are employees of Angelini Pharma. Liliana Dell'Osso, Emi Bondi, Andrea Fagiolini, Paolo Iazzetta and Maurizio Pompili have participated as Principal Investigators in the trial and in meetings as speakers or advisors for Angelini Pharma.

## Data Availability

The data that support the findings of this study are available on request from the corresponding author. The data are not publicly available due to privacy or ethical restrictions.
